# Cyclo­linopeptide B methanol tris­olvate

**DOI:** 10.1107/S1600536811051488

**Published:** 2011-12-07

**Authors:** Gabriele Schatte, Shaunivan Labiuk, Bonnie Li, Peta-Gaye Burnett, Martin Reaney, Pawel Grochulski, Michel Fodje, Jian Yang, Ramaswami Sammynaiken

**Affiliations:** a Saskatchewan Structural Sciences Centre, University of Saskatchewan, Saskatoon, Saskatchewan, Canada S7N 5C9; b Canadian Light Source Inc., University of Saskatchewan, Saskatoon, Saskatchewan, Canada S7N 0X4; c College of Agriculture & Bioresources, University of Saskatchewan, Saskatoon, Saskatchewan, Canada S7N 5A8; d College of Pharmacy and Nutrition, University of Saskatchewan, Saskatoon, Saskatchewan, Canada S7N 5C9

## Abstract

The title compound, C_56_H_83_N_9_O_9_S·3CH_3_OH, is a methanol tris­olvate of the cyclo­linopeptide *cyclo*(Met^1^—Leu^2^—Ile^3^—Pro^4^—Pro^5^—Phe^6^—Phe^7^—Val^8^—Ile^9^) (henceforth referred to as CLP-B), which was isolated from flaxseed oil. All the amino acid residues are in an l-configuration based on the *CORN* rule. The cyclic nona­peptide exhibits eight *trans* peptide bonds and one *cis* peptide bond observed between the two proline residues. The conformation is stabilized by an α-turn and two consecutive β-turns each containing a N—H⋯O hydrogen bond between the carbonyl group O atom of the first residue and the amide group H atom of the fourth (α-turn) or the third residue (β-turns), repectively. In the crystal, the components of the structure are linked by N—H⋯O and O—H⋯O hydrogen bonds into chains parallel to the *a* axis.

## Related literature

For the isolation of cyclo­linopeptides A to B, B to E, F to I and characterization by multi-dimensional NMR spectroscopy, see: Matsumoto *et al.* (2002 ▶), Morita *et al.* (1999 ▶) and Matsumoto *et al.* (2001 ▶), respectively. For the isolation of the related cyclo­linopeptide A and its structure determination by single X-ray diffraction in the presence of different solvates, see: Di Blasio *et al.* (1987 ▶, 1989 ▶); Matsumoto *et al.* (2002 ▶); Quail *et al.* (2009 ▶). For the X-ray single-crystal structure of cyclo­linopeptide K, see: Jadhav *et al.* (2011 ▶). For the synthesis of cyclo­peptides, see: Rovero *et al.* (1991 ▶); Ghadiri *et al.* (1993 ▶). For the immuno-suppressive activity of CLP-A, see: Wieczorek *et al.* (1991 ▶) and for its cytoproctective ability, see: Gaymes *et al.* (1997 ▶). For the biomolecular inter­action with human albumin of CLP-A, see: Rempel *et al.* (2010 ▶). For details of the *CORN* rule, see: Cahn *et al.* (1966 ▶). For details of the absolute structure, see: Flack & Bernardinelli (2000 ▶).
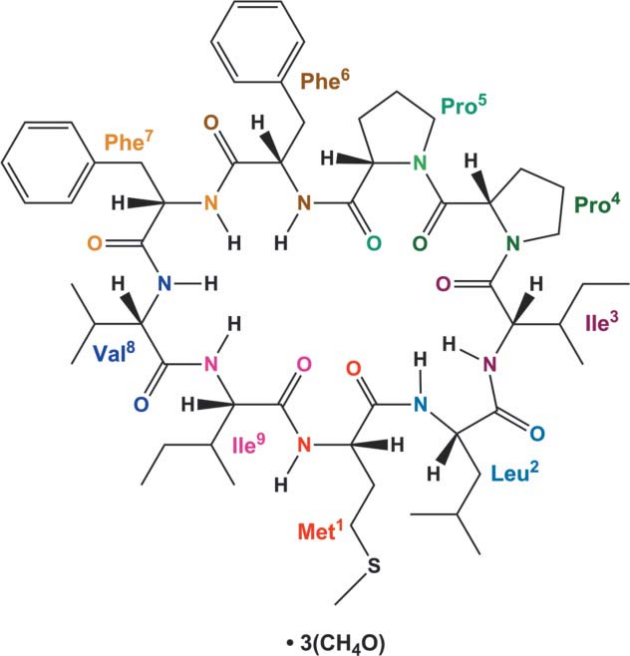


## Experimental

### 

#### Crystal data

C_56_H_83_N_9_O_9_S·3CH_4_O
*M* *_r_* = 1154.50
Monoclinic, 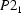

*a* = 10.374 (2) Å
*b* = 19.624 (4) Å
*c* = 15.576 (4) Å
β = 100.0653 (13)°
*V* = 3122.1 (12) Å^3^
*Z* = 2
Synchrotron radiation
λ = 0.68878 Å
μ = 0.12 mm^−1^
*T* = 100 K
0.13 × 0.10 × 0.10 mm

#### Data collection

300mm 16K Rayonix MX300 HE CCD detector with an ACCEL MD2 microdiffractometer
Absorption correction: multi-scan (*SADABS*; Bruker, 2008 ▶) *T*_min_ = 0.985, *T*_max_ = 0.988
177237 measured reflections
15255 independent reflections
13940 reflections with *I* > 2σ(*I*)
*R*_int_ = 0.055

#### Refinement

*R*[*F* ^2^ > 2σ(*F* ^2^)] = 0.048
*wR*(*F* ^2^) = 0.116
*S* = 1.12
15255 reflections
809 parameters
2 restraints
H atoms treated by a mixture of independent and constrained refinement
Δρ_max_ = 0.64 e Å^−3^
Δρ_min_ = −0.34 e Å^−3^
Absolute structure: Flack (1983 ▶), 7080 Friedel pairs
Flack parameter: 0.09 (7)

### 

Data collection: *MXDC*, Macromolecular Crystallography Data Collector (Canadian Light Source, 2007 ▶); cell refinement: *SAINT* (Bruker, 2008 ▶); data reduction: *SAINT*; program(s) used to solve structure: *SIR2004* (Burla *et al.*, 2005 ▶); program(s) used to refine structure: *SHELXL97* (Sheldrick, 2008 ▶); molecular graphics: *CAMERON* (Watkin *et al.*, 1993 ▶) and *SHELXTL* (Sheldrick, 2008 ▶); software used to prepare material for publication: *publCIF* (Westrip, 2010 ▶).

## Supplementary Material

Crystal structure: contains datablock(s) global, I. DOI: 10.1107/S1600536811051488/lh5387sup1.cif

Structure factors: contains datablock(s) I. DOI: 10.1107/S1600536811051488/lh5387Isup2.hkl

Additional supplementary materials:  crystallographic information; 3D view; checkCIF report

## Figures and Tables

**Table 1:** Hydrogen-bond geometry (Å, °)

*D*—H⋯*A*	*D*—H	H⋯*A*	*D*⋯*A*	*D*—H⋯*A*
N1—H1*D*⋯O7	0.87 (3)	2.29 (3)	3.046 (3)	145 (3)
N2—H2*D*⋯O8	0.87 (3)	2.11 (3)	2.923 (3)	155 (3)
N7—H7*D*⋯O3	0.84 (3)	2.18 (3)	2.956 (3)	153 (3)
N8—H8*D*⋯O2^i^	0.83 (3)	2.52 (3)	3.274 (3)	151 (3)
N9—H9*D*⋯O60	0.91 (3)	2.00 (3)	2.896 (3)	169 (3)
N6—H6*D*⋯O70^ii^	0.77 (3)	2.34 (3)	3.071 (3)	159 (3)
O60—H60⋯O1^i^	0.95 (4)	1.79 (4)	2.705 (3)	160 (4)
O70—H70⋯O4^iii^	1.01 (3)	1.91 (2)	2.861 (3)	157 (3)
O80—H80⋯O9^iv^	0.94 (6)	1.86 (6)	2.786 (3)	165 (5)

Symmetry codes: (i) 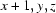; (ii) 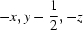; (iii) 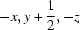; (iv) 
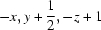
.

**Table 2:** Backbone torsion angles ϕ, ψ, ω and side chain torsion angle χ1 (°)in CLP-B

	ϕ	ψ	ω	χ1
Met^1^	−83.2 (3)	−3.7 (3)	174.6 (2)	−56.0 (3)
Leu^2^	53.4 (3)	42.8 (3)	−172.4 (2)	−48.7 (3)
Ile^3^	−117.2 (3)	99.9 (2)	172.9 (2)	−61.9 (3)
Pro^4^	−76.8 (3)	157.2 (2)	−174.4 (2)	32.3 (2)
Pro^5^	−91.4 (3)	−4.6 (3)	−9.8 (3)	34.2 (2)
Phe^6^	−98.9 (3)	−23.7 (3)	−166.6 (2)	−75.2 (2)
Phe^7^	−116.6 (2)	72.7 (3)	−171.5 (2)	−59.6 (3)
Val^8^	−63.9 (3)	−43.7 (3)	−162.8 (2)	−66.1 (19)
Ile^9^	−69.8 (3)	−19.9 (3)	−177.1 (2)	−155.2 (2)

**Fig. 1.**
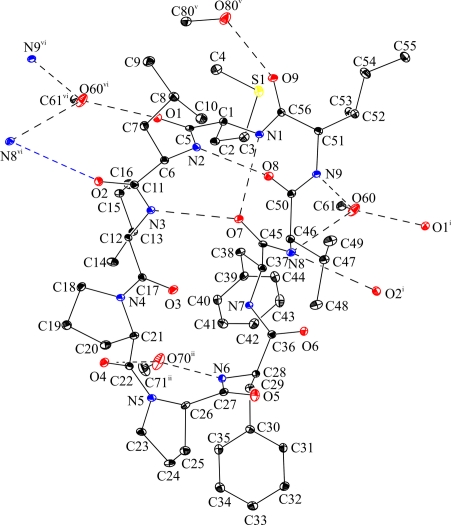
Molecular structure showing the labelling scheme and the inter- and intra-molecular hydrogen bonding. Hydrogen atoms have been omitted for clarity. The non-hydrogen atoms are represented by displacement ellipsoids at the 20% probability level. Symmetry transformations used to generate equivalent atoms: (i) x + 1, y, z; (ii) -x, y - 1/2, -z; (v) -x, y - 1/2, -z + 1; (vi) x - 1, y, z + 1.

**Fig. 2.**
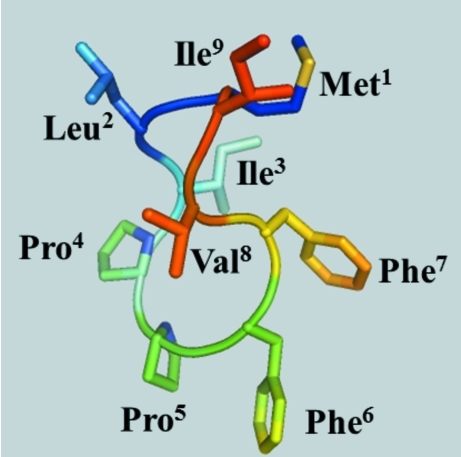
Secondary structure of CLP-B showing the α-turn and the two β-turns. The cartoon representation is traced along the backbone of CLP-B. Hydrogen atoms have been omitted for clarity.

## supplementary crystallographic information

### Comment

Flaxseed (bionamial name: *Linum usitatassimum*) contains mostly triglyceride oil (omega-3 fatty acids, and alpha-linolenic acid *etc.*), to lesser amounts lignans and very small amounts of hydrophobic cyclolinopeptides. These *cyclo* octa-and nonapeptides have attracted significant interest because of their various biological activities, most importantly because of their immuno-suppressive activity (Wieczorek *et al.*, 1991), their cytoprotective ability, their inhibitory activity toward calcium-dependent activation of T-lymphocyte cell division (Gaymes *et al.*, 1997), and their biomolecular interaction with human albumin (Rempel *et al.*, 2010). The structures of nine different cyclolinopeptides (CLP-A to CLP-I) have been elucidated by two-dimensional FT-NMR spectroscopy (Matsumoto *et al.*, 2002; Morita *et al.*, 1999; Matsumoto *et al.*, 2001). Structure determinations of CLP-A with different co-crystallized solvent molecules have been established by single-crystal X-ray diffraction (Di Blasio *et al.*, 1987, 1989; Matsumoto *et al.*, 2002; Quail *et al.* 2009). Recently, the crystal structure of the previously unknown CLP-K has been reported (Jadhav *et al.*, 2011). The crystal structure of *cyclo*(Met^1^—Leu^2^—Ile^3^—Pro^4^—Pro^5^—Phe^6^—Phe^7^—Val^8^—Ile^9^), CLP-B, was determined as part of our ongoing research on the biological activity of flaxseeds from different locations and strains.

All the amino acid residues in CLP-B are in the *L* configuration based on *CORN* rule. The *L* configuration of the amino acid residues in the CLP-B was determined previously using derivative chemistry (Morita *et al.*, 1999). Applying the *Cahn-Ingold-Prelog priority rules* (Cahn *et al.*, 1966), the configuration at the chiral α-C atom of each amino acid residue is *S*. The standard uncertainty *u* = 0.07 at the Flack parameter *x* = 0.09 implies an enantiopure-sufficient inversion-distinguishing power and together with |*x*| < 2*u* one can conclude that the absolute structure is correct (Flack & Bernardinelli, 2000). The cyclolinopeptide exhibits eight *trans* peptide bonds with values for ω ranging from 162.8 (2) to 177.1 (2)° (see Table 2) and one *cis* peptide bond observed between the two proline residues (ω = -9.8 (3)°) (see Table 2). The conformation of the cylic peptide is stabilized by an α-turn and two consecutive β-turns each containing a hydrogen bond between the carbonyl oxygen of the first residue and the amide hydrogen of the fourth (α-turn) or the third residue (β-turn), repectively. The 5→1 NH···O=C contact bond (α-turn) involves the amide group of Phe^7^ and carbonyl group of Ile^3^ with the two *cis* bonded proline residues Pro^4^ and Pro^5^ being part of this α-turn. The α-turn in CLP-B is identical to the one found in CLP-K (Jadhav *et al.*, 2011). In contrast, only one β-turn was located in the crystal structure of CLP-K, which involved the amide group of Ile^3^ and carbonyl group of Ile^9^ (Jadhav *et al.*, 2011). The first β-turn, a 4→1 NH···O=C contact bond, is formed between the amide group of Leu^2^ and the carbonyl group of Val^8^. The second β-turn is observed between the amide group of Met^1^ and the carbonyl group of Phe^7^. The presence of these turns leads to a very twisted conformation of CLP-B with an almost V-shaped part at Pro^5^ as depicted in Fig. 2. The side chains of Met^1^, Leu^2^, Ile^3^, Phe^6^, Phe^7^, Val^8^, Ile^9^ all adopt the *gauche*(+) conformation based on their χ_1_ torsion angles (see Table 2).

The analysis of the conformation of CLP-B in the polar solvent d_6_-DMSO using NMR spectroscopy showed the presence of a γ-turn, 3→1 NH···O═C contact bond, involving the amide group of Val^8^ and carbonyl group of Phe^6^ (Matsumoto *et al.*, 2002). In contrast, this γ-turn is not observed in the solid state structure of CLP-B. In fact, the nitrogen atom of the amide group in Val^8^ and the oxygen atom of carbonyl group in Phe^6^ are separated by 3.670 (3) Å, which exceeds by far the N···O contact distance of 3.07 Å based on the sum of the van der Waals radii for nitrogen and oxygen. The value for the 3→1 NH···O=C contact bond was calculated to be 1.95 Å based on distance geometry (DG) calculations in combination with NMR data (Matsumoto *et al.*, 2002). However, in the crystal structure of CLP-B this distance is 3.06 (3) Å, which is too long for a NH···O═C contact bond.

The CLP-B molecules are linked *via* intermolcular NH···O═C contact bonds. In addition, the CLP-B units are connected *via* one methanol solvent molecule through hydrogen bonds involving a) one carbonyl group of one peptide and the hydrogen atom of the hydroxy group of a methanol molecule and, b) the oxygen atom of the hydroxy group of the same methanol molecule and the hydrogen atoms of the two amide groups of a symmetry related CLP-B molecule (see Table 1). These hydrogen bond interconnections are responsible for the formation of infinite one-dimensional chains parallel to the *a* axis. The remaining two methanol solvent molecules form only one hydrogen bond with either a carbonyl group or an amide group of a symmetry related ClP-B molecule.

### Experimental

Crystals of CLP-B were obtained *via* slow cooling of a saturated solution of CLP-B in methanol. A clear solution of CLP-B (5 mg) in (100 µ*L*) was obtained upon sonicating and heating the CLP-*B*/methanol solvent mixture to 323K. The solution was allowed to reach room temperature. Single small cube-like crystals of CLP-B, suitable for X-ray diffraction, were obtained within two hours.

### Refinement

A suitable single-crystal was removed from the solution, quickly coated with oil (Paratone 8277, Exxon), collected inside a mounted CryoLoop^TM^ (diameter of the nylon fiber: 10 microns; loop diameter 0.1–0.2 mm) and then quickly transferred to the cold stream of the Oxford cryo-jet. The mounted CryoLoop^TM^ had been attached prior to a copper wire (thickness, 0.6 mm; length: 18 mm) attached to a magnetic base using epoxy. Intensity data were collected at 100 K using the beamline 08B1–1 (CMCF-BM; Canadian Light Source) equipped with a ACCEL MD2 microdiffractometer and a 300 mm 16 K Rayonix MX300 HE CCD detector. The wavelength was set to 0.68878 Å and the distance between the detector and the crystal to 150 mm. The initial screening and data collection was performed with the Macromolecular Crystallography Data Collector (MXDC) graphical user interface. A series of data frames at 1° increments of ω were collected. The integrated intensity data were merged and corrected for absorption using *SADABS* (6, 1 harmonics). The final unit-cell parameters are based upon the refinement of the XYZ weighted centroids of 9745 reflections above 20 σ(I) with 4.71° < 2θ < 60.51°.

The C-bound H atoms, with the exception of the α-C-bound H atoms, were geometrically placed (C–H = 0.98–1.00 Å) and refined as riding with *U_iso_*(H) = 1.2*U_eq_*(parent atom). The hydrogen atoms of the amide groups and the the α-C-bound hydrogen atoms were located in the difference Fourier map and were allowed to refine freely. The hydrogen atoms of the hydroxyl groups of the methanol solvent molecules were located in the difference Fourier map and were allowed to refine freely.

### Crystal data

**Table:**

C_56_H_83_N_9_O_9_S·3CH_4_O	*F*(000) = 1248
*M**_r_* = 1154.50	*D*_x_ = 1.228 Mg m^−^^3^
Monoclinic, *P*2_1_	Synchrotron radiation, λ = 0.68878 Å
Hall symbol: P 2yb	Cell parameters from 9745 reflections
*a* = 10.374 (2) Å	θ = 2.4–30.3°
*b* = 19.624 (4) Å	µ = 0.12 mm^−^^1^
*c* = 15.576 (4) Å	*T* = 100 K
β = 100.0653 (13)°	Plate, colourless
*V* = 3122.1 (12) Å^3^	0.13 × 0.10 × 0.10 mm
*Z* = 2	

### Data collection

**Table:**

300mm 16K Rayonix MX300 HE CCD detector with an ACCEL MD2 microdiffractometer	15255 independent reflections
Radiation source: Beamline 08B1-1 at the CLS	13940 reflections with *I* > 2σ(*I*)
double crystal Si(111)	*R*_int_ = 0.055
Detector resolution: 13.8 pixels mm^-1^	θ_max_ = 27.3°, θ_min_ = 1.3°
CCD rotation images, ω scans	*h* = −13→13
Absorption correction: multi-scan (*SADABS*; Bruker, 2008)	*k* = −26→26
*T*_min_ = 0.985, *T*_max_ = 0.988	*l* = −20→20
177237 measured reflections	

### Refinement

**Table:**

Refinement on *F*^2^	Hydrogen site location: inferred from neighbouring sites
Least-squares matrix: full	H atoms treated by a mixture of independent and constrained refinement
*R*[*F*^2^ > 2σ(*F*^2^)] = 0.048	*w* = 1/[σ^2^(*F*_o_^2^) + (0.*P*)^2^ + 3.9515*P*] where *P* = (*F*_o_^2^ + 2*F*_c_^2^)/3
*wR*(*F*^2^) = 0.116	(Δ/σ)_max_ = 0.001
*S* = 1.12	Δρ_max_ = 0.64 e Å^−^^3^
15255 reflections	Δρ_min_ = −0.34 e Å^−^^3^
809 parameters	Extinction correction: *SHELXL97* (Sheldrick, 2008), Fc^*^=kFc[1+0.001xFc^2^λ^3^/sin(2θ)]^-1/4^
2 restraints	Extinction coefficient: 0.0195 (8)
Primary atom site location: structure-invariant direct methods	Absolute structure: Flack (1983), 7080 Friedel pairs
Secondary atom site location: difference Fourier map	Flack parameter: 0.09 (7)

### Special details

**Table:**

Geometry. All e.s.d.'s (except the e.s.d. in the dihedral angle between two l.s. planes) are estimated using the full covariance matrix. The cell e.s.d.'s are taken into account individually in the estimation of e.s.d.'s in distances, angles and torsion angles; correlations between e.s.d.'s in cell parameters are only used when they are defined by crystal symmetry. An approximate (isotropic) treatment of cell e.s.d.'s is used for estimating e.s.d.'s involving l.s. planes.
Refinement. Refinement of *F*^2^ against ALL reflections. The weighted *R*-factor *wR* and goodness of fit *S* are based on *F*^2^, conventional *R*-factors *R* are based on *F*, with *F* set to zero for negative *F*^2^. The threshold expression of *F*^2^ > σ(*F*^2^) is used only for calculating *R*-factors(gt) *etc*. and is not relevant to the choice of reflections for refinement. *R*-factors based on *F*^2^ are statistically about twice as large as those based on *F*, and *R*- factors based on ALL data will be even larger.

### Fractional atomic coordinates and isotropic or equivalent isotropic displacement parameters (Å^2^)

**Table:**

	*x*	*y*	*z*	*U*_iso_*/*U*_eq_	
S1	0.13874 (7)	0.29912 (3)	0.57965 (4)	0.02844 (14)	
O1	−0.19694 (17)	0.11300 (9)	0.52610 (11)	0.0239 (4)	
O2	−0.34076 (16)	−0.02660 (10)	0.41387 (11)	0.0235 (4)	
O3	−0.01559 (18)	−0.02475 (11)	0.22118 (13)	0.0326 (4)	
O4	−0.19208 (17)	−0.09933 (10)	0.03103 (11)	0.0243 (4)	
O5	0.32126 (18)	−0.18176 (9)	0.16363 (13)	0.0292 (4)	
O6	0.47224 (17)	0.01870 (11)	0.21109 (13)	0.0317 (4)	
O7	0.15135 (17)	0.04399 (9)	0.40798 (11)	0.0232 (4)	
O8	0.19776 (16)	−0.04762 (9)	0.59423 (10)	0.0205 (3)	
O9	0.15096 (17)	0.10619 (10)	0.72756 (11)	0.0253 (4)	
N1	0.15582 (19)	0.11428 (10)	0.58285 (12)	0.0174 (4)	
H1D	0.192 (3)	0.0954 (17)	0.542 (2)	0.033 (8)*	
N2	−0.0484 (2)	0.02701 (10)	0.54770 (12)	0.0177 (4)	
H2D	0.034 (3)	0.0177 (16)	0.564 (2)	0.026 (8)*	
N3	−0.1505 (2)	0.00636 (10)	0.37164 (13)	0.0193 (4)	
H3D	−0.061 (3)	0.0128 (16)	0.389 (2)	0.027 (8)*	
N4	−0.18619 (19)	−0.09694 (11)	0.20855 (12)	0.0194 (4)	
N5	−0.00959 (19)	−0.16296 (10)	0.04200 (12)	0.0189 (4)	
N6	0.2166 (2)	−0.08956 (10)	0.09619 (13)	0.0173 (4)	
H6D	0.152 (3)	−0.0764 (13)	0.0689 (17)	0.009 (6)*	
N7	0.2594 (2)	0.01660 (10)	0.22498 (12)	0.0180 (4)	
H7D	0.183 (3)	0.0028 (14)	0.2062 (18)	0.015 (7)*	
N8	0.3453 (2)	−0.00933 (11)	0.41386 (13)	0.0200 (4)	
H8D	0.415 (3)	−0.0093 (16)	0.394 (2)	0.029 (8)*	
N9	0.36326 (19)	0.02755 (10)	0.59635 (12)	0.0168 (4)	
H9D	0.422 (3)	0.0447 (17)	0.565 (2)	0.032 (8)*	
C1	0.0276 (2)	0.14528 (12)	0.56048 (15)	0.0187 (4)	
H1	0.012 (3)	0.1732 (15)	0.6139 (19)	0.022*	
C2	0.0190 (2)	0.18937 (12)	0.47889 (15)	0.0225 (5)	
H2A	0.0262	0.1595	0.4288	0.027*	
H2B	−0.0683	0.2112	0.4670	0.027*	
C3	0.1225 (3)	0.24430 (13)	0.48490 (17)	0.0263 (5)	
H3A	0.1027	0.2731	0.4321	0.032*	
H3B	0.2081	0.2221	0.4842	0.032*	
C4	−0.0273 (3)	0.32857 (14)	0.5739 (2)	0.0333 (6)	
H4A	−0.0607	0.3455	0.5150	0.050*	
H4B	−0.0291	0.3654	0.6161	0.050*	
H4C	−0.0822	0.2908	0.5874	0.050*	
C5	−0.0825 (2)	0.09278 (12)	0.54444 (13)	0.0178 (4)	
C6	−0.1436 (2)	−0.02723 (12)	0.52313 (14)	0.0177 (4)	
H6	−0.093 (3)	−0.0690 (15)	0.5196 (18)	0.021*	
C7	−0.2316 (2)	−0.04099 (13)	0.59055 (15)	0.0219 (5)	
H7A	−0.2821	−0.0832	0.5737	0.026*	
H7B	−0.2951	−0.0031	0.5884	0.026*	
C8	−0.1594 (2)	−0.04872 (13)	0.68467 (15)	0.0237 (5)	
H8	−0.1116	−0.0053	0.7023	0.028*	
C9	−0.2599 (3)	−0.05989 (16)	0.74422 (17)	0.0317 (6)	
H9A	−0.3078	−0.1023	0.7279	0.048*	
H9B	−0.3215	−0.0216	0.7381	0.048*	
H9C	−0.2149	−0.0629	0.8049	0.048*	
C10	−0.0613 (3)	−0.10630 (16)	0.69568 (17)	0.0326 (6)	
H10A	−0.0174	−0.1085	0.7567	0.049*	
H10B	0.0038	−0.0983	0.6582	0.049*	
H10C	−0.1066	−0.1494	0.6793	0.049*	
C11	−0.2228 (2)	−0.01490 (12)	0.43115 (15)	0.0190 (4)	
C12	−0.2052 (2)	0.01339 (13)	0.27855 (14)	0.0191 (4)	
H12	−0.297 (3)	0.0005 (15)	0.2686 (19)	0.023*	
C13	−0.1938 (2)	0.08612 (13)	0.24462 (15)	0.0225 (5)	
H13	−0.0995	0.0998	0.2565	0.027*	
C14	−0.2420 (3)	0.08767 (16)	0.14596 (17)	0.0334 (6)	
H14A	−0.3337	0.0731	0.1332	0.050*	
H14B	−0.1885	0.0568	0.1173	0.050*	
H14C	−0.2346	0.1341	0.1243	0.050*	
C15	−0.2712 (3)	0.13593 (13)	0.29222 (16)	0.0243 (5)	
H15A	−0.3659	0.1267	0.2742	0.029*	
H15B	−0.2485	0.1276	0.3558	0.029*	
C16	−0.2449 (3)	0.21045 (15)	0.27420 (19)	0.0339 (6)	
H16A	−0.1507	0.2195	0.2895	0.051*	
H16B	−0.2922	0.2396	0.3093	0.051*	
H16C	−0.2747	0.2202	0.2122	0.051*	
C17	−0.1293 (2)	−0.03756 (12)	0.23224 (14)	0.0200 (4)	
C18	−0.3208 (2)	−0.11839 (13)	0.21534 (16)	0.0230 (5)	
H18A	−0.3857	−0.0837	0.1900	0.028*	
H18B	−0.3293	−0.1266	0.2768	0.028*	
C19	−0.3384 (2)	−0.18421 (13)	0.16275 (17)	0.0242 (5)	
H19A	−0.3724	−0.1750	0.1004	0.029*	
H19B	−0.3990	−0.2157	0.1855	0.029*	
C20	−0.2001 (2)	−0.21346 (12)	0.17535 (16)	0.0236 (5)	
H20A	−0.1907	−0.2461	0.1284	0.028*	
H20B	−0.1772	−0.2367	0.2324	0.028*	
C21	−0.1144 (2)	−0.15029 (12)	0.17123 (15)	0.0195 (4)	
H21	−0.025 (3)	−0.1559 (16)	0.206 (2)	0.028 (8)*	
C22	−0.1074 (2)	−0.13400 (12)	0.07623 (14)	0.0183 (4)	
C23	−0.0172 (3)	−0.16641 (13)	−0.05321 (15)	0.0233 (5)	
H23A	−0.0089	−0.1205	−0.0780	0.028*	
H23B	−0.1010	−0.1869	−0.0816	0.028*	
C24	0.0990 (3)	−0.21170 (13)	−0.06517 (16)	0.0263 (5)	
H24A	0.0743	−0.2431	−0.1151	0.032*	
H24B	0.1742	−0.1836	−0.0753	0.032*	
C25	0.1328 (3)	−0.25147 (13)	0.02031 (16)	0.0247 (5)	
H25A	0.2262	−0.2649	0.0316	0.030*	
H25B	0.0779	−0.2928	0.0192	0.030*	
C26	0.1030 (2)	−0.20053 (12)	0.08920 (15)	0.0181 (4)	
H26	0.079 (3)	−0.2264 (14)	0.1405 (19)	0.022*	
C27	0.2231 (2)	−0.15589 (12)	0.12020 (14)	0.0189 (4)	
C28	0.3345 (2)	−0.04812 (12)	0.10676 (14)	0.0174 (4)	
H28	0.413 (3)	−0.0782 (15)	0.1174 (18)	0.021*	
C29	0.3369 (2)	−0.00383 (12)	0.02555 (16)	0.0222 (5)	
H29A	0.2502	0.0178	0.0075	0.027*	
H29B	0.4027	0.0328	0.0401	0.027*	
C30	0.3697 (2)	−0.04568 (12)	−0.04882 (14)	0.0201 (4)	
C31	0.4968 (2)	−0.07005 (13)	−0.04352 (15)	0.0233 (5)	
H31	0.5612	−0.0593	0.0058	0.028*	
C32	0.5302 (3)	−0.11006 (15)	−0.10995 (16)	0.0282 (5)	
H32	0.6171	−0.1268	−0.1059	0.034*	
C33	0.4363 (3)	−0.12551 (15)	−0.18226 (16)	0.0286 (6)	
H33	0.4586	−0.1534	−0.2273	0.034*	
C34	0.3104 (3)	−0.10055 (14)	−0.18905 (16)	0.0278 (5)	
H34	0.2467	−0.1106	−0.2391	0.033*	
C35	0.2770 (3)	−0.06054 (13)	−0.12231 (16)	0.0242 (5)	
H35	0.1905	−0.0433	−0.1270	0.029*	
C36	0.3604 (2)	−0.00238 (12)	0.18722 (15)	0.0208 (5)	
C37	0.2786 (2)	0.06758 (12)	0.29396 (14)	0.0196 (4)	
H37	0.365 (3)	0.0789 (15)	0.3052 (19)	0.024*	
C38	0.1985 (3)	0.13154 (13)	0.26756 (16)	0.0239 (5)	
H38A	0.1041	0.1201	0.2555	0.029*	
H38B	0.2135	0.1652	0.3156	0.029*	
C39	0.2388 (2)	0.16159 (13)	0.18688 (17)	0.0232 (5)	
C40	0.1646 (3)	0.15059 (13)	0.10492 (16)	0.0251 (5)	
H40	0.0823	0.1283	0.0998	0.030*	
C41	0.2097 (3)	0.17198 (14)	0.02960 (17)	0.0298 (6)	
H41	0.1580	0.1645	−0.0263	0.036*	
C42	0.3299 (3)	0.20408 (15)	0.03706 (18)	0.0318 (6)	
H42	0.3616	0.2180	−0.0138	0.038*	
C43	0.4036 (3)	0.21588 (16)	0.1182 (2)	0.0358 (7)	
H43	0.4860	0.2380	0.1233	0.043*	
C44	0.3574 (3)	0.19550 (14)	0.19253 (18)	0.0310 (6)	
H44	0.4077	0.2049	0.2483	0.037*	
C45	0.2524 (2)	0.03411 (12)	0.37730 (14)	0.0177 (4)	
C46	0.3195 (2)	−0.05871 (12)	0.47820 (14)	0.0179 (4)	
H46	0.234 (3)	−0.0841 (15)	0.4547 (18)	0.021*	
C47	0.4341 (2)	−0.10966 (13)	0.49627 (15)	0.0229 (5)	
H47	0.5173	−0.0831	0.5105	0.027*	
C48	0.4401 (3)	−0.15251 (14)	0.41486 (18)	0.0305 (6)	
H48A	0.5168	−0.1825	0.4259	0.046*	
H48B	0.4469	−0.1223	0.3657	0.046*	
H48C	0.3605	−0.1801	0.4008	0.046*	
C49	0.4231 (3)	−0.15494 (15)	0.57389 (18)	0.0324 (6)	
H49A	0.3428	−0.1821	0.5609	0.049*	
H49B	0.4204	−0.1265	0.6253	0.049*	
H49C	0.4990	−0.1854	0.5855	0.049*	
C50	0.2898 (2)	−0.02502 (11)	0.56122 (14)	0.0168 (4)	
C51	0.3364 (2)	0.05662 (12)	0.67829 (14)	0.0182 (4)	
H51	0.329 (3)	0.0159 (15)	0.7161 (19)	0.022*	
C52	0.4489 (2)	0.10234 (12)	0.72486 (15)	0.0211 (5)	
H52	0.5333	0.0804	0.7176	0.025*	
C53	0.4466 (2)	0.17364 (13)	0.68547 (17)	0.0249 (5)	
H53A	0.3668	0.1974	0.6943	0.037*	
H53B	0.4480	0.1701	0.6229	0.037*	
H53C	0.5234	0.1992	0.7139	0.037*	
C54	0.4448 (3)	0.10467 (16)	0.82280 (16)	0.0309 (6)	
H54A	0.3667	0.1309	0.8317	0.037*	
H54B	0.4347	0.0576	0.8436	0.037*	
C55	0.5659 (3)	0.13651 (16)	0.87815 (18)	0.0332 (6)	
H55A	0.6442	0.1117	0.8687	0.050*	
H55B	0.5583	0.1340	0.9399	0.050*	
H55C	0.5730	0.1843	0.8613	0.050*	
C56	0.2060 (2)	0.09437 (12)	0.66483 (15)	0.0187 (4)	
O60	0.5397 (2)	0.09999 (10)	0.50357 (16)	0.0394 (5)	
H60	0.632 (4)	0.094 (2)	0.512 (3)	0.059*	
C61	0.5126 (3)	0.16301 (17)	0.46285 (19)	0.0379 (7)	
H61A	0.5391	0.1622	0.4055	0.057*	
H61B	0.5612	0.1988	0.4987	0.057*	
H61C	0.4185	0.1723	0.4558	0.045*	
O70	0.0046 (2)	0.50065 (13)	−0.0002 (2)	0.0543 (7)	
H70	0.087 (4)	0.4745 (17)	−0.003 (3)	0.081*	
C71	0.0460 (4)	0.54449 (19)	0.0714 (3)	0.0583 (11)	
H71A	0.0915	0.5179	0.1208	0.087*	
H71B	−0.0304	0.5669	0.0880	0.087*	
H71C	0.1055	0.5790	0.0550	0.087*	
O80	0.0463 (2)	0.68755 (16)	0.2264 (2)	0.0658 (9)	
H80	−0.009 (5)	0.654 (3)	0.244 (4)	0.099*	
C81	0.1734 (3)	0.66712 (18)	0.2587 (2)	0.0409 (7)	
H81A	0.2347	0.6949	0.2325	0.061*	
H81B	0.1840	0.6191	0.2443	0.061*	
H81C	0.1913	0.6729	0.3222	0.061*	

### Atomic displacement parameters (Å^2^)

**Table:**

	*U*^11^	*U*^22^	*U*^33^	*U*^12^	*U*^13^	*U*^23^
S1	0.0277 (3)	0.0223 (3)	0.0333 (3)	−0.0027 (2)	−0.0004 (3)	0.0035 (3)
O1	0.0205 (8)	0.0256 (9)	0.0256 (9)	0.0034 (7)	0.0036 (7)	−0.0041 (7)
O2	0.0197 (8)	0.0341 (10)	0.0175 (8)	−0.0050 (7)	0.0051 (6)	−0.0004 (7)
O3	0.0229 (9)	0.0368 (11)	0.0421 (11)	−0.0074 (8)	0.0166 (8)	−0.0158 (9)
O4	0.0216 (8)	0.0319 (9)	0.0194 (8)	0.0074 (7)	0.0037 (6)	0.0035 (7)
O5	0.0253 (9)	0.0283 (10)	0.0321 (10)	0.0034 (7)	−0.0001 (7)	0.0098 (8)
O6	0.0176 (9)	0.0407 (11)	0.0368 (10)	−0.0023 (8)	0.0043 (7)	−0.0172 (9)
O7	0.0219 (8)	0.0311 (9)	0.0176 (8)	0.0023 (7)	0.0066 (6)	−0.0019 (7)
O8	0.0209 (8)	0.0239 (8)	0.0184 (8)	−0.0038 (7)	0.0083 (6)	0.0009 (6)
O9	0.0256 (9)	0.0366 (10)	0.0149 (7)	0.0056 (8)	0.0068 (6)	−0.0020 (7)
N1	0.0193 (9)	0.0193 (9)	0.0141 (8)	0.0007 (7)	0.0048 (7)	−0.0003 (7)
N2	0.0174 (9)	0.0188 (9)	0.0168 (9)	−0.0023 (7)	0.0028 (7)	−0.0020 (7)
N3	0.0184 (9)	0.0237 (10)	0.0170 (9)	−0.0015 (8)	0.0067 (7)	−0.0020 (7)
N4	0.0193 (9)	0.0242 (10)	0.0160 (9)	−0.0013 (8)	0.0064 (7)	−0.0058 (7)
N5	0.0224 (10)	0.0206 (9)	0.0145 (9)	0.0016 (8)	0.0055 (7)	−0.0016 (7)
N6	0.0164 (9)	0.0185 (9)	0.0169 (9)	0.0000 (8)	0.0028 (7)	0.0000 (7)
N7	0.0171 (9)	0.0236 (10)	0.0143 (8)	−0.0009 (8)	0.0061 (7)	−0.0023 (7)
N8	0.0212 (10)	0.0242 (10)	0.0166 (9)	−0.0004 (8)	0.0089 (8)	0.0015 (8)
N9	0.0180 (9)	0.0184 (9)	0.0152 (8)	0.0005 (7)	0.0062 (7)	−0.0019 (7)
C1	0.0185 (11)	0.0211 (11)	0.0165 (10)	0.0016 (9)	0.0032 (8)	0.0003 (8)
C2	0.0277 (12)	0.0236 (12)	0.0168 (11)	0.0046 (10)	0.0055 (9)	0.0009 (9)
C3	0.0305 (13)	0.0238 (12)	0.0262 (12)	0.0017 (10)	0.0092 (10)	0.0077 (10)
C4	0.0383 (15)	0.0255 (13)	0.0374 (15)	0.0039 (11)	0.0105 (12)	0.0028 (11)
C5	0.0207 (11)	0.0233 (11)	0.0105 (9)	0.0006 (9)	0.0056 (8)	−0.0025 (8)
C6	0.0188 (10)	0.0190 (10)	0.0161 (10)	−0.0023 (9)	0.0050 (8)	−0.0019 (8)
C7	0.0192 (11)	0.0294 (12)	0.0178 (10)	−0.0040 (9)	0.0048 (8)	0.0017 (9)
C8	0.0262 (12)	0.0284 (12)	0.0177 (11)	−0.0073 (10)	0.0072 (9)	0.0008 (9)
C9	0.0320 (14)	0.0442 (16)	0.0211 (12)	0.0025 (12)	0.0110 (10)	0.0046 (11)
C10	0.0319 (14)	0.0482 (17)	0.0183 (11)	0.0027 (12)	0.0062 (10)	0.0036 (11)
C11	0.0201 (11)	0.0186 (10)	0.0187 (10)	−0.0031 (9)	0.0046 (8)	−0.0037 (8)
C12	0.0172 (10)	0.0275 (12)	0.0131 (9)	−0.0037 (9)	0.0038 (8)	−0.0056 (8)
C13	0.0242 (12)	0.0267 (12)	0.0179 (11)	−0.0007 (9)	0.0072 (9)	−0.0015 (9)
C14	0.0447 (16)	0.0378 (15)	0.0193 (12)	−0.0013 (13)	0.0096 (11)	−0.0020 (11)
C15	0.0285 (13)	0.0259 (12)	0.0196 (11)	0.0005 (10)	0.0071 (9)	−0.0008 (9)
C16	0.0473 (17)	0.0262 (13)	0.0290 (13)	0.0029 (12)	0.0090 (12)	0.0014 (11)
C17	0.0196 (11)	0.0252 (12)	0.0158 (10)	−0.0043 (9)	0.0046 (8)	−0.0026 (9)
C18	0.0197 (11)	0.0239 (12)	0.0276 (12)	−0.0016 (9)	0.0099 (9)	−0.0032 (10)
C19	0.0251 (12)	0.0220 (12)	0.0267 (12)	−0.0034 (9)	0.0078 (10)	−0.0030 (9)
C20	0.0295 (13)	0.0225 (12)	0.0206 (11)	0.0008 (10)	0.0096 (9)	0.0018 (9)
C21	0.0218 (11)	0.0222 (11)	0.0156 (10)	0.0045 (9)	0.0061 (8)	−0.0006 (8)
C22	0.0203 (11)	0.0188 (10)	0.0163 (10)	−0.0022 (8)	0.0045 (8)	−0.0026 (8)
C23	0.0298 (12)	0.0292 (13)	0.0115 (10)	0.0013 (10)	0.0052 (9)	−0.0017 (9)
C24	0.0342 (13)	0.0271 (13)	0.0205 (11)	0.0021 (10)	0.0131 (10)	−0.0044 (9)
C25	0.0294 (13)	0.0204 (11)	0.0260 (12)	−0.0011 (10)	0.0091 (10)	−0.0019 (9)
C26	0.0191 (10)	0.0164 (10)	0.0204 (10)	0.0049 (9)	0.0079 (8)	0.0014 (9)
C27	0.0204 (11)	0.0213 (11)	0.0160 (10)	0.0035 (9)	0.0063 (8)	0.0004 (8)
C28	0.0176 (10)	0.0207 (11)	0.0147 (9)	0.0002 (9)	0.0051 (8)	−0.0010 (8)
C29	0.0252 (12)	0.0210 (11)	0.0218 (11)	−0.0013 (9)	0.0079 (9)	0.0027 (9)
C30	0.0261 (12)	0.0211 (11)	0.0140 (10)	−0.0020 (9)	0.0061 (8)	0.0035 (8)
C31	0.0214 (11)	0.0332 (13)	0.0153 (10)	−0.0021 (10)	0.0036 (9)	0.0033 (9)
C32	0.0264 (12)	0.0397 (15)	0.0203 (12)	0.0038 (11)	0.0091 (10)	0.0010 (11)
C33	0.0342 (14)	0.0384 (15)	0.0154 (11)	−0.0019 (12)	0.0098 (10)	0.0003 (10)
C34	0.0318 (13)	0.0353 (14)	0.0163 (11)	−0.0048 (11)	0.0045 (9)	0.0043 (10)
C35	0.0234 (12)	0.0280 (13)	0.0215 (11)	−0.0031 (10)	0.0045 (9)	0.0073 (9)
C36	0.0220 (11)	0.0210 (11)	0.0192 (11)	0.0032 (9)	0.0029 (9)	−0.0008 (9)
C37	0.0226 (11)	0.0224 (11)	0.0141 (10)	−0.0032 (9)	0.0042 (8)	−0.0008 (8)
C38	0.0278 (13)	0.0230 (12)	0.0229 (12)	0.0023 (10)	0.0104 (10)	0.0006 (9)
C39	0.0242 (12)	0.0204 (11)	0.0257 (12)	0.0050 (9)	0.0064 (9)	0.0044 (9)
C40	0.0252 (12)	0.0253 (12)	0.0244 (12)	−0.0015 (10)	0.0029 (10)	0.0058 (10)
C41	0.0397 (15)	0.0296 (14)	0.0196 (12)	0.0008 (12)	0.0036 (10)	0.0082 (10)
C42	0.0322 (14)	0.0363 (14)	0.0285 (13)	0.0037 (12)	0.0097 (11)	0.0122 (11)
C43	0.0285 (14)	0.0385 (16)	0.0395 (16)	−0.0061 (12)	0.0034 (12)	0.0172 (13)
C44	0.0315 (14)	0.0308 (14)	0.0289 (13)	−0.0062 (11)	0.0003 (11)	0.0066 (11)
C45	0.0210 (11)	0.0173 (10)	0.0158 (10)	−0.0018 (8)	0.0057 (8)	−0.0045 (8)
C46	0.0196 (11)	0.0188 (11)	0.0163 (10)	−0.0027 (9)	0.0059 (8)	0.0005 (8)
C47	0.0254 (12)	0.0237 (12)	0.0208 (11)	0.0027 (9)	0.0074 (9)	−0.0001 (9)
C48	0.0386 (15)	0.0273 (13)	0.0291 (13)	0.0059 (12)	0.0152 (11)	0.0009 (10)
C49	0.0435 (16)	0.0294 (13)	0.0265 (13)	0.0134 (12)	0.0124 (11)	0.0073 (11)
C50	0.0185 (10)	0.0162 (10)	0.0163 (10)	0.0038 (8)	0.0048 (8)	0.0024 (8)
C51	0.0207 (11)	0.0189 (11)	0.0147 (10)	0.0007 (9)	0.0027 (8)	−0.0009 (8)
C52	0.0197 (11)	0.0223 (11)	0.0208 (11)	−0.0009 (9)	0.0023 (8)	−0.0019 (9)
C53	0.0241 (12)	0.0243 (12)	0.0255 (12)	−0.0062 (10)	0.0023 (9)	0.0000 (10)
C54	0.0329 (14)	0.0396 (15)	0.0190 (11)	−0.0124 (12)	0.0015 (10)	0.0007 (11)
C55	0.0300 (14)	0.0441 (16)	0.0233 (13)	−0.0046 (12)	−0.0011 (10)	−0.0050 (11)
C56	0.0175 (10)	0.0202 (11)	0.0180 (10)	−0.0020 (8)	0.0022 (8)	−0.0028 (8)
O60	0.0289 (10)	0.0249 (10)	0.0703 (15)	−0.0027 (8)	0.0254 (10)	−0.0009 (10)
C61	0.0386 (16)	0.0470 (17)	0.0295 (14)	0.0136 (14)	0.0099 (12)	0.0007 (13)
O70	0.0355 (12)	0.0381 (12)	0.093 (2)	−0.0076 (10)	0.0226 (13)	−0.0271 (13)
C71	0.057 (2)	0.0386 (18)	0.067 (2)	−0.0053 (17)	−0.0233 (19)	0.0066 (17)
O80	0.0290 (12)	0.079 (2)	0.092 (2)	0.0073 (12)	0.0186 (13)	0.0611 (17)
C81	0.0309 (15)	0.055 (2)	0.0384 (16)	0.0066 (14)	0.0109 (12)	0.0220 (15)

### Geometric parameters (Å, °)

**Table:**

S1—C4	1.804 (3)	C21—H21	1.00 (3)
S1—C3	1.810 (3)	C23—C24	1.534 (3)
O1—C5	1.237 (3)	C23—H23A	0.9900
O2—C11	1.228 (3)	C23—H23B	0.9900
O3—C17	1.247 (3)	C24—C25	1.530 (4)
O4—C22	1.231 (3)	C24—H24A	0.9900
O5—C27	1.230 (3)	C24—H24B	0.9900
O6—C36	1.227 (3)	C25—C26	1.537 (3)
O7—C45	1.241 (3)	C25—H25A	0.9900
O8—C50	1.243 (3)	C25—H25B	0.9900
O9—C56	1.236 (3)	C26—C27	1.530 (3)
N1—C56	1.350 (3)	C26—H26	1.01 (3)
N1—C1	1.449 (3)	C28—C36	1.527 (3)
N1—H1D	0.87 (3)	C28—C29	1.538 (3)
N2—C5	1.337 (3)	C28—H28	1.00 (3)
N2—C6	1.457 (3)	C29—C30	1.507 (3)
N2—H2D	0.87 (3)	C29—H29A	0.9900
N3—C11	1.356 (3)	C29—H29B	0.9900
N3—C12	1.468 (3)	C30—C35	1.391 (3)
N3—H3D	0.93 (3)	C30—C31	1.392 (3)
N4—C17	1.329 (3)	C31—C32	1.390 (3)
N4—C21	1.463 (3)	C31—H31	0.9500
N4—C18	1.480 (3)	C32—C33	1.388 (4)
N5—C22	1.351 (3)	C32—H32	0.9500
N5—C26	1.466 (3)	C33—C34	1.381 (4)
N5—C23	1.473 (3)	C33—H33	0.9500
N6—C27	1.353 (3)	C34—C35	1.394 (4)
N6—C28	1.454 (3)	C34—H34	0.9500
N6—H6D	0.77 (3)	C35—H35	0.9500
N7—C36	1.341 (3)	C37—C45	1.521 (3)
N7—C37	1.456 (3)	C37—C38	1.522 (3)
N7—H7D	0.84 (3)	C37—H37	0.91 (3)
N8—C45	1.337 (3)	C38—C39	1.512 (3)
N8—C46	1.452 (3)	C38—H38A	0.9900
N8—H8D	0.83 (3)	C38—H38B	0.9900
N9—C50	1.341 (3)	C39—C44	1.387 (4)
N9—C51	1.468 (3)	C39—C40	1.388 (4)
N9—H9D	0.91 (3)	C40—C41	1.401 (3)
C1—C5	1.526 (3)	C40—H40	0.9500
C1—C2	1.527 (3)	C41—C42	1.383 (4)
C1—H1	1.03 (3)	C41—H41	0.9500
C2—C3	1.513 (4)	C42—C43	1.378 (4)
C2—H2A	0.9900	C42—H42	0.9500
C2—H2B	0.9900	C43—C44	1.387 (4)
C3—H3A	0.9900	C43—H43	0.9500
C3—H3B	0.9900	C44—H44	0.9500
C4—H4A	0.9800	C46—C50	1.531 (3)
C4—H4B	0.9800	C46—C47	1.541 (3)
C4—H4C	0.9800	C46—H46	1.03 (3)
C6—C7	1.532 (3)	C47—C49	1.521 (3)
C6—C11	1.541 (3)	C47—C48	1.532 (3)
C6—H6	0.98 (3)	C47—H47	1.0000
C7—C8	1.533 (3)	C48—H48A	0.9800
C7—H7A	0.9900	C48—H48B	0.9800
C7—H7B	0.9900	C48—H48C	0.9800
C8—C10	1.510 (4)	C49—H49A	0.9800
C8—C9	1.528 (3)	C49—H49B	0.9800
C8—H8	1.0000	C49—H49C	0.9800
C9—H9A	0.9800	C51—C56	1.524 (3)
C9—H9B	0.9800	C51—C52	1.548 (3)
C9—H9C	0.9800	C51—H51	1.00 (3)
C10—H10A	0.9800	C52—C53	1.526 (3)
C10—H10B	0.9800	C52—C54	1.534 (3)
C10—H10C	0.9800	C52—H52	1.0000
C12—C17	1.529 (3)	C53—H53A	0.9800
C12—C13	1.534 (3)	C53—H53B	0.9800
C12—H12	0.98 (3)	C53—H53C	0.9800
C13—C14	1.531 (3)	C54—C55	1.528 (4)
C13—C15	1.536 (3)	C54—H54A	0.9900
C13—H13	1.0000	C54—H54B	0.9900
C14—H14A	0.9800	C55—H55A	0.9800
C14—H14B	0.9800	C55—H55B	0.9800
C14—H14C	0.9800	C55—H55C	0.9800
C15—C16	1.523 (4)	O60—C61	1.396 (4)
C15—H15A	0.9900	O60—H60	0.95 (4)
C15—H15B	0.9900	C61—H61A	0.9800
C16—H16A	0.9800	C61—H61B	0.9800
C16—H16B	0.9800	C61—H61C	0.9800
C16—H16C	0.9800	O70—C71	1.415 (4)
C18—C19	1.523 (3)	O70—H70	1.01 (3)
C18—H18A	0.9900	C71—H71A	0.9800
C18—H18B	0.9900	C71—H71B	0.9800
C19—C20	1.526 (3)	C71—H71C	0.9800
C19—H19A	0.9900	O80—C81	1.386 (4)
C19—H19B	0.9900	O80—H80	0.94 (6)
C20—C21	1.533 (3)	C81—H81A	0.9800
C20—H20A	0.9900	C81—H81B	0.9800
C20—H20B	0.9900	C81—H81C	0.9800
C21—C22	1.528 (3)		
			
C4—S1—C3	101.41 (13)	C24—C25—H25A	111.1
C56—N1—C1	122.33 (19)	C26—C25—H25A	111.1
C56—N1—H1D	115 (2)	C24—C25—H25B	111.1
C1—N1—H1D	120 (2)	C26—C25—H25B	111.1
C5—N2—C6	122.2 (2)	H25A—C25—H25B	109.0
C5—N2—H2D	117 (2)	N5—C26—C27	113.81 (19)
C6—N2—H2D	121 (2)	N5—C26—C25	102.53 (19)
C11—N3—C12	122.7 (2)	C27—C26—C25	109.92 (18)
C11—N3—H3D	119.5 (19)	N5—C26—H26	111.4 (16)
C12—N3—H3D	117.5 (19)	C27—C26—H26	109.6 (16)
C17—N4—C21	120.4 (2)	C25—C26—H26	109.4 (16)
C17—N4—C18	127.5 (2)	O5—C27—N6	123.1 (2)
C21—N4—C18	112.15 (19)	O5—C27—C26	119.2 (2)
C22—N5—C26	126.96 (19)	N6—C27—C26	117.7 (2)
C22—N5—C23	120.3 (2)	N6—C28—C36	116.46 (18)
C26—N5—C23	112.30 (18)	N6—C28—C29	110.90 (19)
C27—N6—C28	120.4 (2)	C36—C28—C29	108.48 (19)
C27—N6—H6D	118 (2)	N6—C28—H28	109.6 (16)
C28—N6—H6D	120 (2)	C36—C28—H28	101.0 (16)
C36—N7—C37	119.8 (2)	C29—C28—H28	109.9 (16)
C36—N7—H7D	121.1 (19)	C30—C29—C28	111.25 (19)
C37—N7—H7D	118.7 (19)	C30—C29—H29A	109.4
C45—N8—C46	120.9 (2)	C28—C29—H29A	109.4
C45—N8—H8D	117 (2)	C30—C29—H29B	109.4
C46—N8—H8D	122 (2)	C28—C29—H29B	109.4
C50—N9—C51	118.21 (19)	H29A—C29—H29B	108.0
C50—N9—H9D	117 (2)	C35—C30—C31	119.2 (2)
C51—N9—H9D	124 (2)	C35—C30—C29	122.0 (2)
N1—C1—C5	112.67 (19)	C31—C30—C29	118.7 (2)
N1—C1—C2	110.83 (19)	C32—C31—C30	120.5 (2)
C5—C1—C2	108.24 (19)	C32—C31—H31	119.8
N1—C1—H1	107.1 (16)	C30—C31—H31	119.8
C5—C1—H1	105.9 (16)	C33—C32—C31	119.7 (2)
C2—C1—H1	112.1 (16)	C33—C32—H32	120.1
C3—C2—C1	114.5 (2)	C31—C32—H32	120.1
C3—C2—H2A	108.6	C34—C33—C32	120.3 (2)
C1—C2—H2A	108.6	C34—C33—H33	119.8
C3—C2—H2B	108.6	C32—C33—H33	119.8
C1—C2—H2B	108.6	C33—C34—C35	119.8 (2)
H2A—C2—H2B	107.6	C33—C34—H34	120.1
C2—C3—S1	115.94 (18)	C35—C34—H34	120.1
C2—C3—H3A	108.3	C30—C35—C34	120.4 (2)
S1—C3—H3A	108.3	C30—C35—H35	119.8
C2—C3—H3B	108.3	C34—C35—H35	119.8
S1—C3—H3B	108.3	O6—C36—N7	122.9 (2)
H3A—C3—H3B	107.4	O6—C36—C28	117.9 (2)
S1—C4—H4A	109.5	N7—C36—C28	119.0 (2)
S1—C4—H4B	109.5	N7—C37—C45	108.13 (19)
H4A—C4—H4B	109.5	N7—C37—C38	111.67 (19)
S1—C4—H4C	109.5	C45—C37—C38	114.59 (19)
H4A—C4—H4C	109.5	N7—C37—H37	108.2 (18)
H4B—C4—H4C	109.5	C45—C37—H37	105.0 (18)
O1—C5—N2	123.8 (2)	C38—C37—H37	108.9 (19)
O1—C5—C1	118.8 (2)	C39—C38—C37	109.22 (19)
N2—C5—C1	117.4 (2)	C39—C38—H38A	109.8
N2—C6—C7	114.02 (18)	C37—C38—H38A	109.8
N2—C6—C11	110.88 (18)	C39—C38—H38B	109.8
C7—C6—C11	112.32 (19)	C37—C38—H38B	109.8
N2—C6—H6	106.3 (17)	H38A—C38—H38B	108.3
C7—C6—H6	106.3 (17)	C44—C39—C40	118.4 (2)
C11—C6—H6	106.4 (17)	C44—C39—C38	120.3 (2)
C6—C7—C8	115.00 (19)	C40—C39—C38	121.0 (2)
C6—C7—H7A	108.5	C39—C40—C41	120.8 (2)
C8—C7—H7A	108.5	C39—C40—H40	119.6
C6—C7—H7B	108.5	C41—C40—H40	119.6
C8—C7—H7B	108.5	C42—C41—C40	119.6 (2)
H7A—C7—H7B	107.5	C42—C41—H41	120.2
C10—C8—C9	110.0 (2)	C40—C41—H41	120.2
C10—C8—C7	112.9 (2)	C43—C42—C41	120.1 (3)
C9—C8—C7	108.9 (2)	C43—C42—H42	120.0
C10—C8—H8	108.3	C41—C42—H42	120.0
C9—C8—H8	108.3	C42—C43—C44	120.0 (3)
C7—C8—H8	108.3	C42—C43—H43	120.0
C8—C9—H9A	109.5	C44—C43—H43	120.0
C8—C9—H9B	109.5	C43—C44—C39	121.2 (3)
H9A—C9—H9B	109.5	C43—C44—H44	119.4
C8—C9—H9C	109.5	C39—C44—H44	119.4
H9A—C9—H9C	109.5	O7—C45—N8	122.0 (2)
H9B—C9—H9C	109.5	O7—C45—C37	123.4 (2)
C8—C10—H10A	109.5	N8—C45—C37	114.6 (2)
C8—C10—H10B	109.5	N8—C46—C50	112.54 (19)
H10A—C10—H10B	109.5	N8—C46—C47	109.24 (19)
C8—C10—H10C	109.5	C50—C46—C47	112.86 (19)
H10A—C10—H10C	109.5	N8—C46—H46	109.2 (16)
H10B—C10—H10C	109.5	C50—C46—H46	102.8 (16)
O2—C11—N3	123.8 (2)	C47—C46—H46	110.0 (16)
O2—C11—C6	121.6 (2)	C49—C47—C48	110.9 (2)
N3—C11—C6	114.5 (2)	C49—C47—C46	111.2 (2)
N3—C12—C17	105.22 (19)	C48—C47—C46	110.3 (2)
N3—C12—C13	112.82 (19)	C49—C47—H47	108.1
C17—C12—C13	111.64 (19)	C48—C47—H47	108.1
N3—C12—H12	109.3 (17)	C46—C47—H47	108.1
C17—C12—H12	109.2 (17)	C47—C48—H48A	109.5
C13—C12—H12	108.6 (17)	C47—C48—H48B	109.5
C14—C13—C12	109.5 (2)	H48A—C48—H48B	109.5
C14—C13—C15	111.4 (2)	C47—C48—H48C	109.5
C12—C13—C15	110.34 (19)	H48A—C48—H48C	109.5
C14—C13—H13	108.5	H48B—C48—H48C	109.5
C12—C13—H13	108.5	C47—C49—H49A	109.5
C15—C13—H13	108.5	C47—C49—H49B	109.5
C13—C14—H14A	109.5	H49A—C49—H49B	109.5
C13—C14—H14B	109.5	C47—C49—H49C	109.5
H14A—C14—H14B	109.5	H49A—C49—H49C	109.5
C13—C14—H14C	109.5	H49B—C49—H49C	109.5
H14A—C14—H14C	109.5	O8—C50—N9	121.9 (2)
H14B—C14—H14C	109.5	O8—C50—C46	118.5 (2)
C16—C15—C13	113.4 (2)	N9—C50—C46	119.55 (19)
C16—C15—H15A	108.9	N9—C51—C56	111.59 (18)
C13—C15—H15A	108.9	N9—C51—C52	112.97 (19)
C16—C15—H15B	108.9	C56—C51—C52	111.15 (19)
C13—C15—H15B	108.9	N9—C51—H51	104.2 (16)
H15A—C15—H15B	107.7	C56—C51—H51	108.4 (16)
C15—C16—H16A	109.5	C52—C51—H51	108.2 (16)
C15—C16—H16B	109.5	C53—C52—C54	111.8 (2)
H16A—C16—H16B	109.5	C53—C52—C51	112.67 (19)
C15—C16—H16C	109.5	C54—C52—C51	109.49 (19)
H16A—C16—H16C	109.5	C53—C52—H52	107.6
H16B—C16—H16C	109.5	C54—C52—H52	107.6
O3—C17—N4	121.5 (2)	C51—C52—H52	107.6
O3—C17—C12	120.7 (2)	C52—C53—H53A	109.5
N4—C17—C12	117.6 (2)	C52—C53—H53B	109.5
N4—C18—C19	103.23 (18)	H53A—C53—H53B	109.5
N4—C18—H18A	111.1	C52—C53—H53C	109.5
C19—C18—H18A	111.1	H53A—C53—H53C	109.5
N4—C18—H18B	111.1	H53B—C53—H53C	109.5
C19—C18—H18B	111.1	C55—C54—C52	114.2 (2)
H18A—C18—H18B	109.1	C55—C54—H54A	108.7
C18—C19—C20	103.2 (2)	C52—C54—H54A	108.7
C18—C19—H19A	111.1	C55—C54—H54B	108.7
C20—C19—H19A	111.1	C52—C54—H54B	108.7
C18—C19—H19B	111.1	H54A—C54—H54B	107.6
C20—C19—H19B	111.1	C54—C55—H55A	109.5
H19A—C19—H19B	109.1	C54—C55—H55B	109.5
C19—C20—C21	103.27 (19)	H55A—C55—H55B	109.5
C19—C20—H20A	111.1	C54—C55—H55C	109.5
C21—C20—H20A	111.1	H55A—C55—H55C	109.5
C19—C20—H20B	111.1	H55B—C55—H55C	109.5
C21—C20—H20B	111.1	O9—C56—N1	122.6 (2)
H20A—C20—H20B	109.1	O9—C56—C51	120.2 (2)
N4—C21—C22	110.41 (19)	N1—C56—C51	117.18 (19)
N4—C21—C20	102.88 (18)	C61—O60—H60	107 (3)
C22—C21—C20	109.62 (19)	O60—C61—H61A	109.5
N4—C21—H21	110.7 (18)	O60—C61—H61B	109.5
C22—C21—H21	110.7 (17)	H61A—C61—H61B	109.5
C20—C21—H21	112.3 (18)	O60—C61—H61C	109.5
O4—C22—N5	121.1 (2)	H61A—C61—H61C	109.5
O4—C22—C21	121.3 (2)	H61B—C61—H61C	109.5
N5—C22—C21	117.4 (2)	C71—O70—H70	101.3 (18)
N5—C23—C24	104.12 (19)	O70—C71—H71A	109.5
N5—C23—H23A	110.9	O70—C71—H71B	109.5
C24—C23—H23A	110.9	H71A—C71—H71B	109.5
N5—C23—H23B	110.9	O70—C71—H71C	109.5
C24—C23—H23B	110.9	H71A—C71—H71C	109.5
H23A—C23—H23B	109.0	H71B—C71—H71C	109.5
C25—C24—C23	104.82 (19)	C81—O80—H80	107 (3)
C25—C24—H24A	110.8	O80—C81—H81A	109.5
C23—C24—H24A	110.8	O80—C81—H81B	109.5
C25—C24—H24B	110.8	H81A—C81—H81B	109.5
C23—C24—H24B	110.8	O80—C81—H81C	109.5
H24A—C24—H24B	108.9	H81A—C81—H81C	109.5
C24—C25—C26	103.41 (19)	H81B—C81—H81C	109.5
			
C56—N1—C1—C5	−83.2 (3)	N5—C26—C27—O5	177.0 (2)
C56—N1—C1—C2	155.4 (2)	C25—C26—C27—O5	−68.6 (3)
N1—C1—C2—C3	−56.0 (3)	N5—C26—C27—N6	−4.6 (3)
C5—C1—C2—C3	179.94 (19)	C25—C26—C27—N6	109.7 (2)
C1—C2—C3—S1	−51.7 (3)	C27—N6—C28—C36	−98.9 (3)
C4—S1—C3—C2	−53.6 (2)	C27—N6—C28—C29	136.4 (2)
C6—N2—C5—O1	4.8 (3)	N6—C28—C29—C30	−75.2 (2)
C6—N2—C5—C1	−172.38 (18)	C36—C28—C29—C30	155.7 (2)
N1—C1—C5—O1	178.92 (19)	C28—C29—C30—C35	109.0 (3)
C2—C1—C5—O1	−58.2 (3)	C28—C29—C30—C31	−71.0 (3)
N1—C1—C5—N2	−3.7 (3)	C35—C30—C31—C32	−1.4 (4)
C2—C1—C5—N2	119.2 (2)	C29—C30—C31—C32	178.6 (2)
C5—N2—C6—C7	−74.5 (3)	C30—C31—C32—C33	0.3 (4)
C5—N2—C6—C11	53.4 (3)	C31—C32—C33—C34	0.9 (4)
N2—C6—C7—C8	−48.7 (3)	C32—C33—C34—C35	−1.0 (4)
C11—C6—C7—C8	−175.9 (2)	C31—C30—C35—C34	1.3 (4)
C6—C7—C8—C10	−59.3 (3)	C29—C30—C35—C34	−178.7 (2)
C6—C7—C8—C9	178.2 (2)	C33—C34—C35—C30	−0.1 (4)
C12—N3—C11—O2	−4.0 (4)	C37—N7—C36—O6	3.9 (4)
C12—N3—C11—C6	172.9 (2)	C37—N7—C36—C28	−171.5 (2)
N2—C6—C11—O2	−140.2 (2)	N6—C28—C36—O6	160.7 (2)
C7—C6—C11—O2	−11.3 (3)	C29—C28—C36—O6	−73.4 (3)
N2—C6—C11—N3	42.8 (3)	N6—C28—C36—N7	−23.7 (3)
C7—C6—C11—N3	171.7 (2)	C29—C28—C36—N7	102.3 (2)
C11—N3—C12—C17	−117.2 (2)	C36—N7—C37—C45	−116.6 (2)
C11—N3—C12—C13	120.8 (2)	C36—N7—C37—C38	116.4 (2)
N3—C12—C13—C14	175.2 (2)	N7—C37—C38—C39	−59.6 (3)
C17—C12—C13—C14	56.9 (3)	C45—C37—C38—C39	177.1 (2)
N3—C12—C13—C15	−61.9 (3)	C37—C38—C39—C44	−74.1 (3)
C17—C12—C13—C15	179.9 (2)	C37—C38—C39—C40	100.0 (3)
C14—C13—C15—C16	−68.6 (3)	C44—C39—C40—C41	1.3 (4)
C12—C13—C15—C16	169.6 (2)	C38—C39—C40—C41	−172.8 (2)
C21—N4—C17—O3	2.2 (4)	C39—C40—C41—C42	0.4 (4)
C18—N4—C17—O3	−178.2 (2)	C40—C41—C42—C43	−1.1 (4)
C21—N4—C17—C12	−174.4 (2)	C41—C42—C43—C44	0.1 (5)
C18—N4—C17—C12	5.3 (4)	C42—C43—C44—C39	1.7 (5)
N3—C12—C17—O3	−76.7 (3)	C40—C39—C44—C43	−2.3 (4)
C13—C12—C17—O3	46.0 (3)	C38—C39—C44—C43	171.8 (3)
N3—C12—C17—N4	99.9 (2)	C46—N8—C45—O7	14.8 (3)
C13—C12—C17—N4	−137.4 (2)	C46—N8—C45—C37	−162.8 (2)
C17—N4—C18—C19	169.4 (2)	N7—C37—C45—O7	−104.9 (3)
C21—N4—C18—C19	−10.9 (3)	C38—C37—C45—O7	20.4 (3)
N4—C18—C19—C20	30.8 (2)	N7—C37—C45—N8	72.7 (2)
C18—C19—C20—C21	−39.5 (2)	C38—C37—C45—N8	−162.1 (2)
C17—N4—C21—C22	−76.8 (3)	C45—N8—C46—C50	−63.9 (3)
C18—N4—C21—C22	103.5 (2)	C45—N8—C46—C47	169.9 (2)
C17—N4—C21—C20	166.3 (2)	N8—C46—C47—C49	170.4 (2)
C18—N4—C21—C20	−13.4 (2)	C50—C46—C47—C49	44.4 (3)
C19—C20—C21—N4	32.3 (2)	N8—C46—C47—C48	−66.1 (3)
C19—C20—C21—C22	−85.2 (2)	C50—C46—C47—C48	167.9 (2)
C26—N5—C22—O4	174.6 (2)	C51—N9—C50—O8	2.7 (3)
C23—N5—C22—O4	−13.3 (3)	C51—N9—C50—C46	−177.10 (19)
C26—N5—C22—C21	−9.8 (3)	N8—C46—C50—O8	136.5 (2)
C23—N5—C22—C21	162.3 (2)	C47—C46—C50—O8	−99.3 (2)
N4—C21—C22—O4	−27.3 (3)	N8—C46—C50—N9	−43.7 (3)
C20—C21—C22—O4	85.4 (3)	C47—C46—C50—N9	80.5 (3)
N4—C21—C22—N5	157.2 (2)	C50—N9—C51—C56	−69.8 (3)
C20—C21—C22—N5	−90.2 (2)	C50—N9—C51—C52	164.1 (2)
C22—N5—C23—C24	−171.6 (2)	N9—C51—C52—C53	79.7 (2)
C26—N5—C23—C24	1.6 (3)	C56—C51—C52—C53	−46.6 (3)
N5—C23—C24—C25	20.4 (3)	N9—C51—C52—C54	−155.2 (2)
C23—C24—C25—C26	−34.1 (3)	C56—C51—C52—C54	78.4 (2)
C22—N5—C26—C27	−91.4 (3)	C53—C52—C54—C55	−65.8 (3)
C23—N5—C26—C27	96.0 (2)	C51—C52—C54—C55	168.6 (2)
C22—N5—C26—C25	150.0 (2)	C1—N1—C56—O9	−6.1 (4)
C23—N5—C26—C25	−22.7 (2)	C1—N1—C56—C51	174.6 (2)
C24—C25—C26—N5	34.2 (2)	N9—C51—C56—O9	160.7 (2)
C24—C25—C26—C27	−87.1 (2)	C52—C51—C56—O9	−72.2 (3)
C28—N6—C27—O5	11.7 (3)	N9—C51—C56—N1	−19.9 (3)
C28—N6—C27—C26	−166.60 (19)	C52—C51—C56—N1	107.2 (2)

### Hydrogen-bond geometry (Å, °)

**Table:**

*D*—H···*A*	*D*—H	H···*A*	*D*···*A*	*D*—H···*A*
N1—H1D···O7	0.87 (3)	2.29 (3)	3.046 (3)	145 (3)
N2—H2D···O8	0.87 (3)	2.11 (3)	2.923 (3)	155 (3)
N7—H7D···O3	0.84 (3)	2.18 (3)	2.956 (3)	153 (3)
N8—H8D···O2^i^	0.83 (3)	2.52 (3)	3.274 (3)	151 (3)
N9—H9D···O60	0.91 (3)	2.00 (3)	2.896 (3)	169 (3)
N6—H6D···O70^ii^	0.77 (3)	2.34 (3)	3.071 (3)	159 (3)
O60—H60···O1^i^	0.95 (4)	1.79 (4)	2.705 (3)	160 (4)
O70—H70···O4^iii^	1.01 (3)	1.91 (2)	2.861 (3)	157 (3)
O80—H80···O9^iv^	0.94 (6)	1.86 (6)	2.786 (3)	165 (5)

Symmetry codes: (i) *x*+1, *y*, *z*; (ii) −*x*, *y*−1/2, −*z*; (iii) −*x*, *y*+1/2, −*z*; (iv) −*x*, *y*+1/2, −*z*+1.

### Table 2 Backbone torsion angles φ, ψ, ω and side chain torsion angle χ1 (°)in CLP-B

**Table:**

	φ	ψ	ω	χ1
Met^1^	-83.2 (3)	-3.7 (3)	174.6 (2)	-56.0 (3)
Leu^2^	53.4 (3)	42.8 (3)	-172.4 (2)	-48.7 (3)
Ile^3^	-117.2 (3)	99.9 (2)	172.9 (2)	-61.9 (3)
Pro^4^	-76.8 (3)	157.2 (2)	-174.4 (2)	32.3 (2)
Pro^5^	-91.4 (3)	-4.6 (3)	-9.8 (3)	34.2 (2)
Phe^6^	-98.9 (3)	-23.7 (3)	-166.6 (2)	-75.2 (2)
Phe^7^	-116.6 (2)	72.7 (3)	-171.5 (2)	-59.6 (3)
Val^8^	-63.9 (3)	-43.7 (3)	-162.8 (2)	-66.1 (19)
Ile^9^	-69.8 (3)	-19.9 (3)	-177.1 (2)	-155.2 (2)
